# Sex Differences in Hypertrophic Cardiomyopathy: Interaction With Genetics and Environment

**DOI:** 10.1007/s11897-021-00526-x

**Published:** 2021-09-03

**Authors:** Alexandra Butters, Neal K. Lakdawala, Jodie Ingles

**Affiliations:** 1 Centre for Population, Genomics, Garvan Institute of Medical Research and UNSW Sydney, Sydney, Australia; 2 Centre for Population Genomics, Murdoch Children’s Research Institute, Melbourne, Australia; 3 Centenary Institute and Faculty of Medicine and Health, The University of Sydney, Sydney, Australia; 4 Cardiovascular Medicine, Brigham and Women’s Hospital, Harvard Medical School, Boston, USA; 5 Department of Cardiology, Royal Prince Alfred Hospital, Sydney, Australia

**Keywords:** Hypertrophic cardiomyopathy, Sex, Genetics, Environment

## Abstract

**Purpose of Review:**

We explore the sex-specific interaction of genetics and the environment on the clinical course and outcomes of hypertrophic cardiomyopathy (HCM).

**Recent Findings:**

Women account for approximately one-third of patients in specialist HCM centres and reported in observational studies. As a result, evidence informing clinical guideline recommendations is based predominantly on risk factors and outcomes seen in men. However, disease progression appears to be different between the sexes. Women present at a more advanced stage of disease, are older at diagnosis, have higher symptom burden, carry greater risk for heart failure and are at greater risk of mortality compared to men. Women are more likely to be gene-positive, while men are more likely to be gene-negative. The risk of sudden cardiac death and access to specialised care do not differ between the sexes.

**Summary:**

Reporting sex-disaggregated results is essential to identify the mechanisms leading to sex differences in HCM.

## Introduction

Hypertrophic cardiomyopathy (HCM) is the most common inherited heart disease affecting 0.5% of the adult population [1]. It is a primary myocardial disorder characterised by left ventricular hypertrophy, which is not explained by abnormal loading conditions such as hypertension [2]. Clinical heterogeneity is common with many individuals experiencing minimal symptoms, while others can develop heart failure, atrial fibrillation or sudden cardiac death (SCD). Traditionally HCM has been considered a monogenic disease caused by variants in genes encoding the cardiac sarcomere [3]. Despite ongoing research over three decades, the underlying genetic basis of HCM is incompletely understood. At least 40% of HCM patients have no family history and no causative variant identified [4, 5]. In some cases, patients remain gene-elusive despite evidence for heritability, and there is growing awareness that a proportion of HCM is due to a complex interplay between genetic and environmental factors. Indeed, results from two recent genome-wide association studies in large HCM cohorts suggest that common variants account for an important proportion of heritability (SNP heritability ~ 30%), suggesting a role for genetic and non-genetic factors in penetrance and expression [6, 7].

**Fig. 1:**
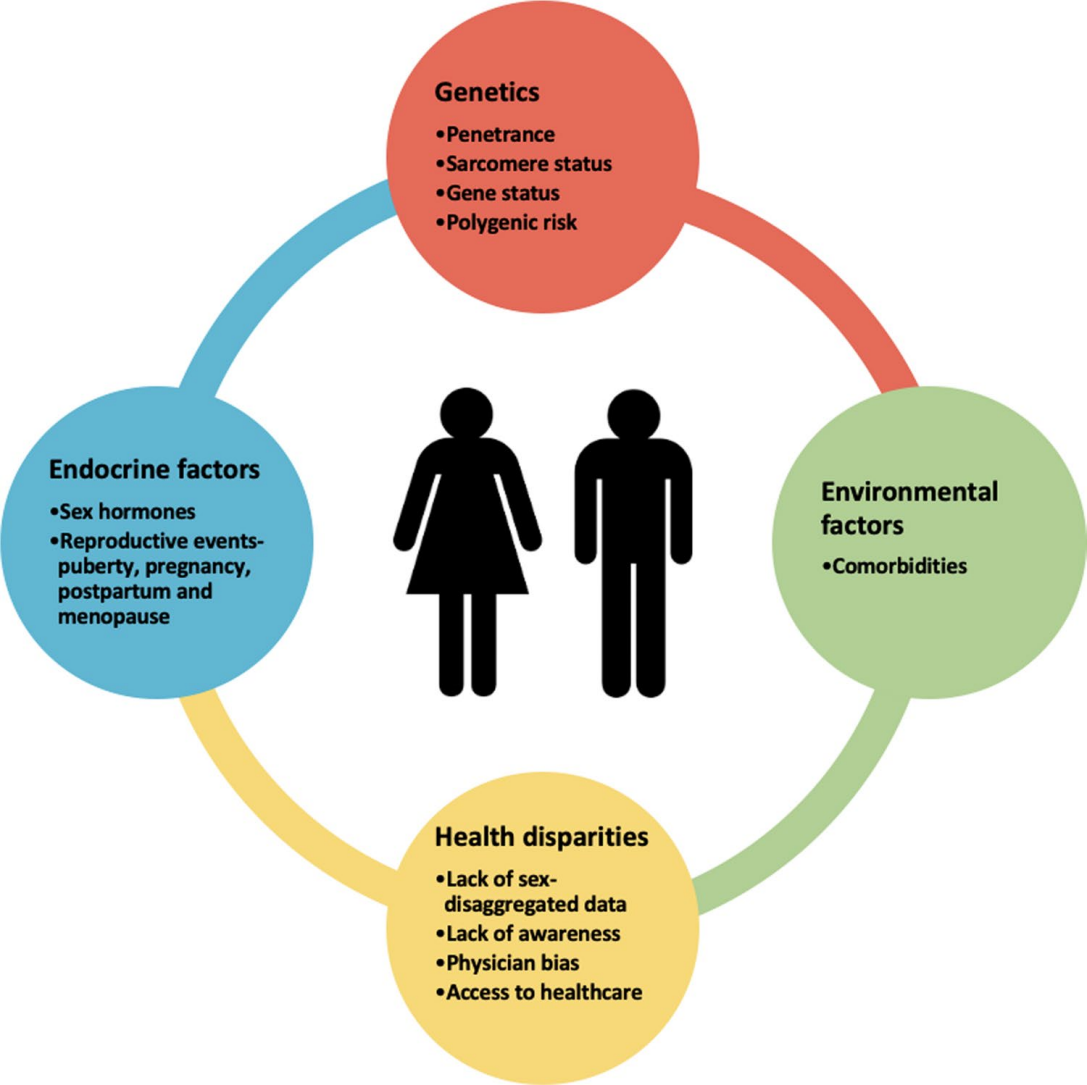
Complex relationship of factors that determine the sex-related differences in hypertrophic cardiomyopathy

Sex-based differences in HCM have gained attention in recent years. There is overwhelming evidence that sex is vital in explaining some of the clinical heterogeneity seen among HCM patients (Fig. 1). Despite an autosomal dominant inheritance pattern, males consistently account for approximately 60% of published cohorts [8–15]. Indeed, the penetrance of sarcomere variants may be lower in women; however, diagnostic biases and under-recognition of HCM may also play a role. We know women are older at the time of diagnosis, have a higher prevalence of an obstructive phenotype, worse diastolic function and more severe heart failure symptoms at presentation [8–15]. Women are also more likely to be sarcomere variant positive (sarcomere-positive), i.e. have a disease-causing variant identified in one of the eight key sarcomere genes as opposed to men who are more likely to be sarcomere variant negative (sarcomere-negative), i.e. no family history and no causative variant identified [4]. Under-representation of females in cohort studies means that the evidence informing clinical guideline recommendations is based predominantly on risk factors and outcomes seen in men. We aim to explore the sex-specific interaction of genetics and the environment on the clinical course and outcomes of HCM.

## Sex Differences in Natural History and Clinical Course

### Prevalence

HCM is typically caused by variants in genes encoding proteins of the sarcomere and inherited as an autosomal dominant trait. Therefore, the prevalence of HCM in the general population is expected to be equal among the sexes. However, studies frequently report an overrepresentation of males, ranging from 55 to 70% [8–15]. This has not changed over the last three decades, or since the first significant publication, examining sex differences in HCM (37% vs. 38%, *p* = 0.34) [14]. Over the life course, the male predominance persists across all age groups until 60 years of age when females become the more prevalent group [9, 10, 16]. It is unknown if this finding represents decreased disease penetrance in women or reflects differences in clinical recognition, slower disease progression due to the protection of sex hormones or lack of sex-specific diagnostic criteria.

### Age at Diagnosis

Women have consistently been shown to be older at diagnosis than men, ranging from 6 to 13 years [9, 10, 12–14]. Furthermore, the age of diagnosis differs by sex depending on genotype. A recent study of *n* = 5873 patients (38% female) by the Sarcomeric Human Cardiomyopathy registry (SHaRe) found that the age of diagnosis varies by sex, genotype and disease gene. Sex-specific differences in age at diagnosis were more pronounced in sarcomere-negative HCM than sarcomere-positive HCM (7.1 years older vs. 3.6 years older) [14]. Differences also existed depending on the gene status of sarcomere-positive patients. Those with a disease-causing variant in *MYBPC3* or a thin-filament gene (i.e. *TNNT2*, *TNNI3*, *TPM1*, *ACTC*) were 4.8 and 6.7 years older than men at diagnosis, respectively (*p* < 0.02). Age at diagnosis was comparable for patients with variants in *MYH7*. Using age at diagnosis as a proxy for penetrance, the authors suggested that sex does not appear to modify the penetrance of *MYH7* variants to the same degree as variants in other sarcomere genes. Age at diagnosis has been explored in those with left ventricular outflow tract obstruction. Olivotto et al. studied *n* = 969 HCM patients (41% female) and found the delay to diagnosis even more striking with female patients, who were up to 14 years older than their male counterparts [9].

### Symptoms at Initial Evaluation

Women are more likely to present with heart failure symptoms, particularly exertional dyspnoea, fatigue, palpitations and chest pain and New York Heart Association (NYHA) functional class III to IV, compared to men [8–15]. Determinants of initial HCM diagnosis differ between men and women [9, 13]. A multi-centre study of *n* = 2123 HCM patients (38% female) [13] found that heart failure symptoms led to diagnosis more commonly in women (51% versus 41% in men, *p* = 0.02), while in men, the diagnosis often followed a routine medical assessment that identified an abnormal electrocardiogram or heart murmur (32% versus 23%, *p* < 0.01). There was no difference in the proportion of patients diagnosed due to clinical screening for family history. Specifically, 73% of women were symptomatic in NYHA functional classes II to IV, characteristically with exertional dyspnoea or fatigue, with or without chest pain compared to 53% of men (*p* < 0.001).

### Morphology

While measurement of maximum left ventricular (LV) wall thickness, LV cavity size and left atrial diameter is often statistically greater in males, these are typically not clinically significant differences [9, 12–14]. The female heart is smaller than males after correction for body surface area (BSA) [17–21], and corrected SHaRe registry data showed that women have a greater maximal LV wall thickness, LV cavity size and left atrial diameter [14]. HCM diagnostic criteria do not currently account for the fact that women have relatively smaller hearts than men [2, 22]. This means that women require relatively more hypertrophy to reach current diagnostic criteria of at least 15-mm maximal LV wall thickness. Smaller heart size may explain the delay in recognising the disease and advanced symptoms seen in women. No difference exists in the prevalence of severe hypertrophy (LV wall thickness ≥ 30 mm) [9, 12, 13].

Despite similar degrees of hypertrophy, women more commonly demonstrate dynamic LV outflow tract obstruction (≥ 30 mmHg) than men [9, 12–14]. In a study of *n* = 2123 patients (38% female), Rowin et al. found that women more commonly demonstrated dynamic LV outflow tract obstruction caused by mitral valve systolic anterior motion than did men (65% versus 57%; *p* < 0.001). LVOT obstruction was present most commonly at rest (45% versus 31% in men; *p* < 0.001) and less frequently provoked with stress exercise echocardiography (20% versus 26% in men; *p* < 0.001) [13]. Similarly, Geske et al. showed that women have a higher LVOT gradient when compared with men [36 (IQR 10–81) vs. 23 (IQR 0–61) mmHg] and were more likely to demonstrate resting obstruction when compared with men (54% vs. 46%, *p* < 0.0001). Consistent with these results, women had more mitral regurgitation (moderate to high in 28% vs. 18%, *p* < 0.0001) [12]. This finding is likely related to the smaller cavity size in females. This hypothesis is supported by the SHaRe registry, which found that after controlling for LV end-diastolic diameter, the sex-based difference in LVOT obstruction did not persist (*p* = 0.17) [14].

Echocardiographic assessment of diastolic function using spectral and tissue Doppler imaging was available for a subset of patients in two studies [12, 14]. Both studies found relatively impaired diastolic function in women. Data from the SHaRe registry highlight that although the peak early diastolic septal tissue Doppler velocity (*e*′) was lower in women (6.1 ± 2.6 versus 7.0 ± 2.7, *p* < 0.0001), the peak velocity of early diastolic inflow (*E* wave; 83.5 ± 32.7 versus 75.0 ± 23.7, *p* < 0.0001) and the ratio of *E*/*e*′ (15.6 ± 9.2 versus 11.9 ± 6.3, *p* < 0.0001) were higher in women [14]. However, these differences were not significant after controlling for age and hypertension. LVOT obstruction is a powerful predictor of adverse outcomes due to heart failure. Therefore, the more frequent occurrence of obstruction in women likely contributes to their more significant adverse outcomes.

## Clinical Outcomes

### Heart Failure

Female patients with HCM have a greater risk than male patients of progression to NYHA III–IV symptoms and heart failure. Recent data showed that incident heart failure was 87% more common in women even after controlling for obstruction, systolic dysfunction, hypertension and age (HR 1.87, CI 1.48–2.32, *p* < 0.001). The authors hypothesised that the increased burden of heart failure in women may be related to LVOT obstruction and diastolic function differences. Systolic dysfunction is infrequent in HCM and not more common in women [14, 23]. Understanding contributors to the higher frequency of heart failure in women is essential to improve overall outcomes.

Rowin et al. showed that during follow-up, women are more likely to develop advanced drug-refractory heart failure (NYHA class III/IV) (53% versus 35% men; *p* < 0.001). This diagnosis occurred on average 6 years later than in males. However, women were still relatively young, with 48% of women < 50 years of age at onset (range, 14–49) [13]. They also found that despite greater use of beta-blockers, verapamil and disopyramide in women, the rate of progression to heart failure was higher in women (4.8%/year versus 3.4%/year; HR 1.6, CI 1.2–2.1, *p* < 0.001). Advanced heart failure symptoms secondary to outflow obstruction were reported in the same number of women and men.

Consistent with the higher prevalence of heart failure, women were more likely to undergo septal reduction therapy, including septal myectomy and alcohol septal ablation. Improvement to NYHA I–II symptoms following these procedures has been reported to be similar between the sexes [24]. Of the 283 women and 347 male patients who underwent septal myectomy with NYHA III–IV symptoms, 94% of female patients and 97% of male improved to NYHA I–II by the end of follow-up (*p* = 0.18). Septal reduction therapy is therefore just as successful in controlling LVOT obstruction in both women and men and has likely resulted in a reduction in mortality from heart failure associated with HCM.

#### Arrhythmias

Patients with HCM are at high risk of developing ventricular and atrial arrhythmias. Female sex was not a predictor of SCD events (i.e. SCD, resuscitated out-of-hospital cardiac arrest or appropriate ICD shock for ventricular tachycardia or ventricular fibrillation) (HR 0.69, CI 0.51–0.94, *p* < 0.05) or atrial fibrillation (AF) (HR 0.72, CI 0.60–0.87, *p* < 0.001) [5]. The prevalence of SCD events is similar between the sexes in numerous studies. One study found that the occurrence of SCD events is 5% (0.9%/year) in women and 6% (0.8%/year) in men (HR 0.92; CI 0.6–1.5, *p* = 0.73) [13]. Accordingly, the investigators also found no difference in primary prevention ICD insertions between the sexes. Despite women being older at diagnosis, the age at ICD implantation also did not differ.

The prevalence of symptomatic AF is similar between women and men at baseline [9, 12–14]. Lakdawala et al. found the incidence of AF to be 22% in both sexes. However, in a multivariate model controlling for age, left atrial diameter and hypertension, they found women to be at a moderately increased risk of incident AF (HR 1.21, CI 1.01–1.46, *p* = 0.04).

#### Mortality

Several studies have been conducted in HCM patients to determine whether women with HCM have poorer outcomes than men with HCM [9, 11–15, 25]. Geske et al. recently reported outcomes of HCM patients attending the Mayo Clinic. This study had a large sample size of 3673 adult patients (45% female) and the most extended follow-up so far [10.9 years (IQR 7.4–16.2 years)]. Women had a significantly higher incidence of all-cause mortality than both men with HCM and the general population. Furthermore, female sex was associated with all-cause mortality in multivariable and propensity-matched analyses (HR 1.17, 95% CI 1.07–1.25; *p* < 0.001).

A multi-centre report by the European HCM Outcome Investigators’ collaboration included 4893 patients (36% women) with a follow-up of 6.2 years (IQR, 3.1–9.8 years) [25] showed that excess mortality was greater among women than men (HR 1.19, 95% CI:1.06–1.30; *p* = 0.007). This persisted into the later decades of life, whereas mortality rates in men aged more than 65 years were similar to the general population. The authors suggest that this may be related to increased heart failure death rates (HR 1.44, 95% CI:1.25–1.59; *p* < 0.001). The SHaRe registry, *n* = 5873 patients (38% female) and median of 7.7 years (IQR, 3.1–15.4 years) follow-up, reported that all-cause mortality is 50% higher in women after controlling for age, sarcomere status, systolic dysfunction (LVEF < 50%) and left atrial diameter [HR, 1.50 (95% CI, 1.13–1.99), *p* < 0.01] [14]. Of all deaths, 43% were HCM-related (caused by heart failure, sudden death or stroke), with a similar frequency of causes in women and men. This study provides further insights into HCM-specific mechanisms contributing to decreased survival in women. It is not a reflection of the greater prevalence of sarcomeric HCM as the difference persisted after controlling for genotype. Excess mortality is also not due to stroke or sudden death. The investigators, therefore, suggest that excess heart failure is the most plausible mechanism.

Other studies have not found any difference in all-cause mortality. In a 2005 study of 969 patients with HCM (41% female), female sex was associated with progression to severe heart failure and death from heart failure or stroke [1.50 (1.11–2.00), *p* < 0.001], but not all-cause mortality [9]. Similarly, a recent study of 2123 patients (38% female) referred to the Tuft’s HCM Institute between 2001 and 2016 [13] showed that age-adjusted all-cause mortality did not differ between women and men (HR 1.32, 95% CI 0.92–1.91, *p* = 0.13). Both studies report small samples and may lack statistical power to see an effect.

## Genetic Basis of HCM

There is growing awareness that a proportion of HCM is due to a complex interplay between genetic and environmental factors. Incomplete penetrance and variable expressivity are hallmark features of the disease, and recent work has begun to identify the role of common genetic variants and non-genetic/environmental factors in contributing to the phenotype.

### Sarcomere-Positive HCM

HCM is traditionally a Mendelian disease inherited in an autosomal dominant manner. Eight sarcomere genes have the most robust gene-disease association (*MYBPC3*, *MYH7*, *TNNT2*, *TNNI3*, *TPM1*, *ACTC*, *MYL2* and *MYL3*) [3].

Women are more likely to have a disease-causing variant identified and are therefore more likely to be sarcomere-positive. Sex differences related to genetics have recently been comprehensively reported, with genetic testing performed in 3788 (65%) patients. Females were 17% more likely to have a causative sarcomere variant (*p* < 0.001). The distribution of disease genes, however, did not vary between the sexes. As expected, the most commonly involved genes were *MYBPC3* and *MYH7* [14]. A study by Terauchi et al. investigated the penetrance of MYBPC3 mutations in 61 patients from 28 families [26]. They found that disease penetrance in those ≤ 40 years of age was 92% in males and 67% in females. Another retrospective study of sarcomere variant carriers found that male sex and an abnormal ECG are associated with a higher risk of developing HCM [27]. The underlying mechanisms for sex-based differences in the penetrance and expression of sarcomeric HCM are not well understood.

### Sarcomere-Negative HCM

**Fig. 2:**
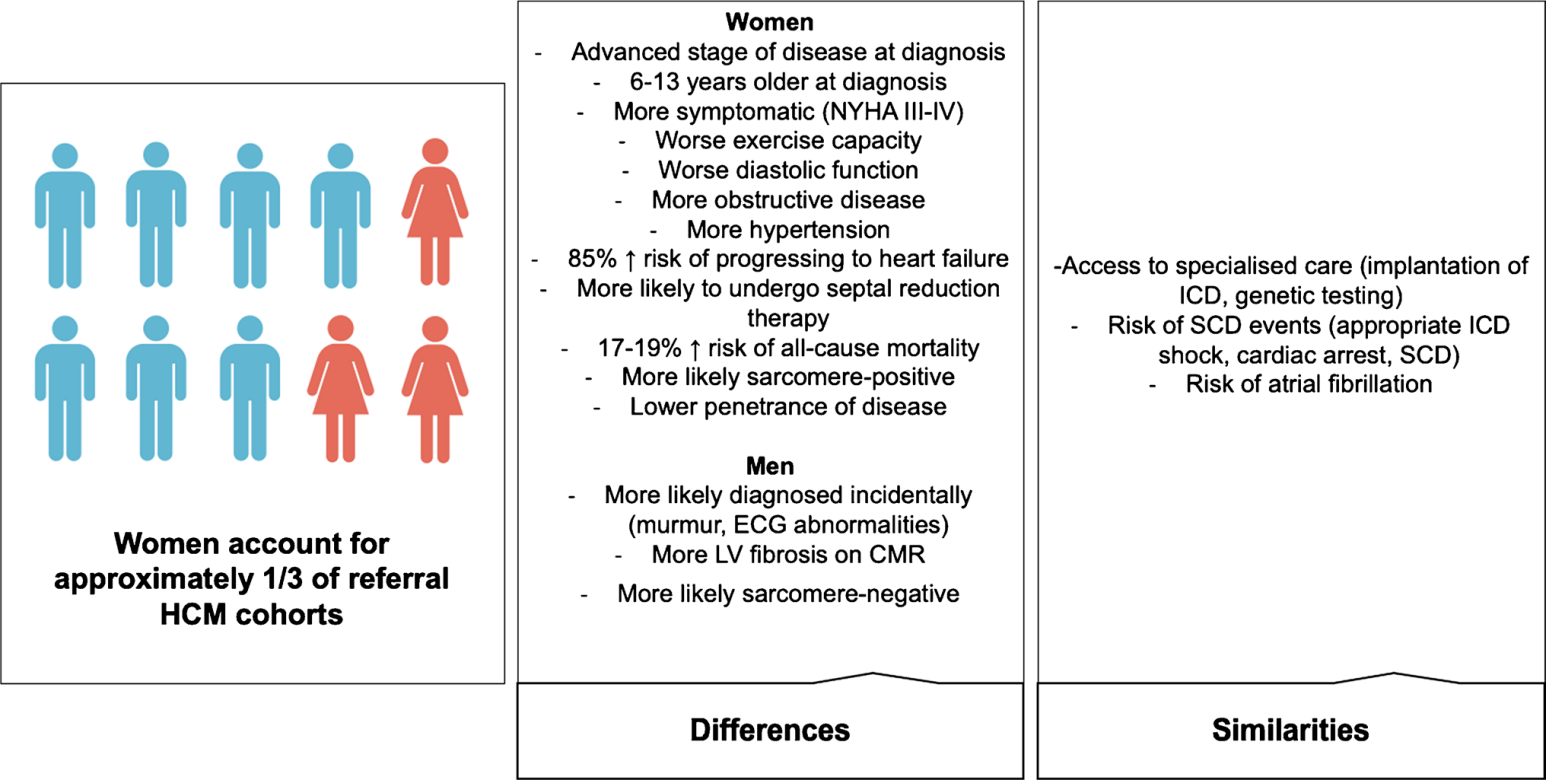
Sex differences in clinical profile, genotype and outcomes in hypertrophic cardiomyopathy

A subgroup of patients with HCM have no genetic variant identified despite comprehensive genetic testing, and family screening does not identify an affected relative even after decades of clinical surveillance [4, 5]. This subgroup has a different clinical course compared with sarcomere-positive HCM, being typically male, diagnosed later in life, with milder LV hypertrophy and more likely to have a pre-existing diagnosis of hypertension [4–7]. This sub-group of patients likely have an underlying complex disease aetiology. Recent genome-wide association studies performed in large international cohorts of patients with HCM have shown SNP heritability up to 34%, and individuals with sarcomere-negative HCM are more likely to be in the extreme polygenic risk score (PRS) distributions [4] (Fig. 2).

## Non-genetic Risk Factors in HCM

### Hypertension

Based on the high population prevalence, many HCM patients will develop hypertension in their lifetime. Hypertension at diagnosis with HCM is an independent predictor of outcomes, irrespective of ethnicity, sex or age (HR 2.02, 95% CI 1.05 to 3.88, *p* = 0.036) [28]. Women are, however, more likely to carry a prior diagnosis of hypertension compared to men (49% vs. 43%, *p* = 0.0010) [12]. Hypertension has been found to be a predictor of sarcomere-negative status (OR, 2.80; 95% CI, 1.57–5.00; *p* = 0.0005) [4], and more recently a one standard deviation increase in diastolic blood pressure (11.3 mmHg) was shown to confer a four-fold risk of HCM (OR 3.93, 95% CI 2.86–5.41, *p* = 3.74 × 10^−16^) independent of other factors [6]. The authors noted that this is more than double the risk typically observed for other diseases associated with diastolic blood pressure [29–33] and raises the possibility that sarcomere-negative HCM may constitute an exaggerated response to hypertension in genetically susceptible individuals. Given known differences in frequency of sarcomere variants and prevalence of hypertension between sexes, how this effect might be different for males and females should be explored.

### Obesity

Obesity is a significant public health problem and a known risk factor for cardiovascular disease. Notwithstanding the limitations of using body mass index (BMI) to define obesity, this problem is widespread among adult patients with HCM, with > 70% being pre-obese with a BMI > 25 and > 30% obese with a BMI > 30 [34–36]. Obesity is common in paediatric patients, with approximately 30% having a BMI in the 99th percentile for age and sex [37]. Excess weight is associated with increased left ventricular hypertrophy and mass and is a prognostic factor for rapid clinical progression and worsening of heart failure symptoms [34, 36]. LV obstruction is more common in obese patients and observed in more than 50% with body mass index > 30 [35]. Obese patients were less likely to carry a sarcomere gene variant; however, obesity increases the risk of significant outcomes in both sarcomere-positive and sarcomere-negative patients. Little is known about sex-specific differences in obesity in HCM. Lakdawala et al. found no difference in BMI between males and females (*p* = 0.72) [14]; however, Geske et al. found a statistically significant, but very small difference (28.3 ± 6.7 female versus 28.9 ± 5.0 male, *p* = 0.0053) [12]. Due to the influence of obesity on the progression of HCM, more research is required to identify if differences between men and women exist.

### Obstructive Sleep Apnoea

Obstructive sleep apnoea (OSA) is exceedingly prevalent in HCM, affecting 32–70% of patients [38, 39]. This wide range is likely due to differences in the definition of sleep-disordered breathing. Patients with OSA are older, have more hypertension and have greater limiting symptoms and exercise capacity [38–41]. OSA has also been associated with a greater prevalence of AF (40% vs. 11%, *p* = 0.005) [39] and apnoea-hypopnea index is associated with an increased risk of non-sustained VT (OR 1.07; 95% CI 1.02–1.12; *p* = 0.001) [40]. Sex-related differences in OSA have not yet been investigated, likely due to the small sample size of the published cohorts. More research is needed to investigate if sex differences exist.

### Pregnancy

Pregnancy places substantial burden on the cardiovascular system, triggering increased circulating blood volume, stroke volume and heart rate [42]. Pregnancy in HCM is well tolerated, however, does still carry maternal and foetal risks [43–47]. A meta-analysis of data from 9 cohorts, including 237 women and 408 pregnancies, demonstrated that most pregnancies in women with HCM are uneventful [44]. The risk of maternal mortality is low occurring in only 0.5% of cases. Complications or worsening symptoms occurred in 29% of cases and included heart failure in up to 30% and arrhythmias in up to 48%. A study of 276 women with cardiovascular disease, including 8% cardiomyopathies, found that the risk of adverse outcomes during delivery is very low (3–4%) with no differing risk between natural delivery and caesarean section [42]. In terms of foetal outcomes, the risk of premature birth is increased occurring in 26% of births. Foetal mortality is comparable to the general population [44]. The impact of parity is largely unknown in HCM; however, there is an association between the number of pregnancies and the risk of cardiovascular disease [48]. This warrants further investigation in HCM cohorts.

## Health Disparities

**Fig. 3:**
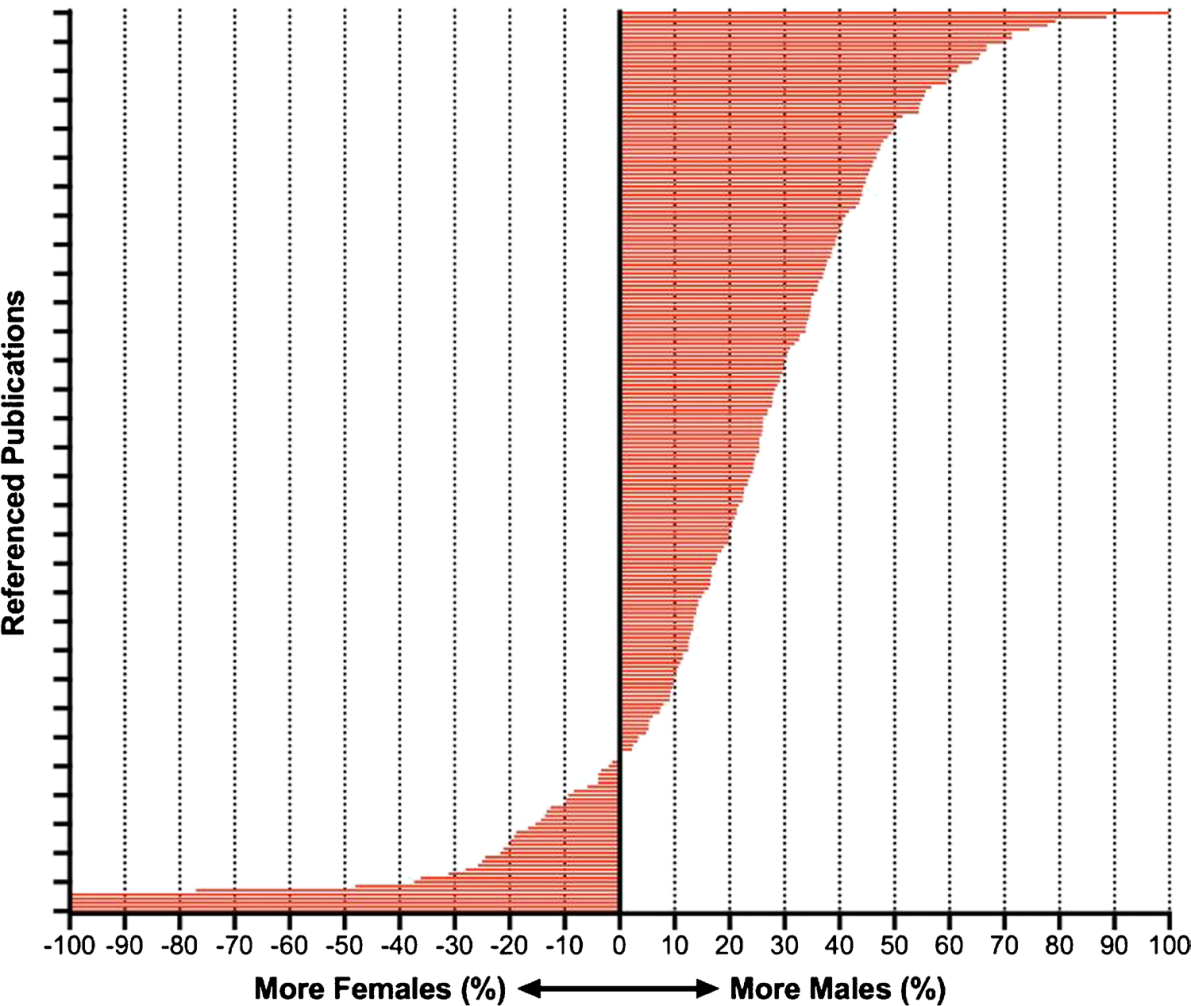
Sex distribution of original research studies used to inform clinical recommendations in the 2020 AHA 2020 AHA/ACC guideline for the diagnosis and treatment of patients with hypertrophic cardiomyopathy [2]

Health disparities likely play an important role in the differences observed between males and females. However, little is known about the impact of health disparities concerning sex in HCM. Women have been consistently underrepresented in published studies, leaving a paucity of pre-specified sex-disaggregated evidence (i.e. evidence where sub-group analyses are specified by sex before data collection enabling the collection, analysis and reporting of data separately for men and women). This has resulted in clinical guideline recommendations informed largely by male-dominated datasets. Figure 3 shows the proportion of women and men in original research studies used to form 2020 AHA/ACC guideline recommendations to diagnose and treat patients with hypertrophic cardiomyopathy. Of the 219 studies, 13 provided no breakdown of sex within their cohorts, despite controlling sex in their analysis. Five studies with 100% female participants were specifically investigating pregnancy. Women tended to be over-represented in studies relating to septal reduction therapies. The single study with 100% males consisted of *n* = 10 males and investigated VT ablation in HCM. While this is possibly representative of disease prevalence, ensuring relatively equal numbers of males and females in studies is necessary to identify why these differences exist. It is crucial that we more often report sex-disaggregated results for all studies on HCM, even if this is not the study’s primary focus. It is also essential to highlight that women from non-European populations are likely to have more severe outcomes and will be even more underrepresented in the literature. These data can inform research and patient care to the benefit of both sexes.

Despite this lack of female data in clinical guidelines, limited disparities have been identified in access to HCM-based management once women present to a specialised HCM clinic. Women are just as likely to have an implantable cardioverter defibrillator (ICD) [9, 12–14]. Additionally, despite the older age at diagnosis, women undergo ICD implantation at a similar age to men [13]. ICD use in HCM differs from population-based studies, which have consistently found women to be less likely to receive an ICD, even after adjustment for known clinical confounders, including age and comorbidities [49, 50]. No bias exists between the sexes in regards to genetic testing [14]. Rates of septal reduction therapy do, however, appear to differ. Women are more likely to undergo alcohol septal ablation and septal myectomy [51, 52]. This likely relates to the increased age at diagnosis and higher prevalence of obstruction and heart failure symptoms in females.

## Physiological or Social Causes?

The causes of sex-specific differences in HCM are primarily unknown. Several mechanisms have been suggested relating to underlying physiological differences and societal factors. The endocrine effects of sex hormones are the most widely accepted physiological mechanism for sex differences in a range of cardiovascular diseases [53–55]. The contribution of hormonal and reproductive factors to HCM risk is still incompletely understood. There is limited evidence in humans and animal models that sex hormones may delay development of the HCM phenotype or its clinical manifestations. Oestrogens have a protective role in hypertrophy [56], while exposure of cardiomyocytes to androgens may result in hypertrophy [57]. This suggests that pre-menopausal women may be protected against developing HCM compared to men. One study suggested that the high number of younger women reported (< 50 years) with heart failure is perhaps suggestive that menopause is unlikely to play a part in HCM [13].

Delayed diagnosis and worse symptoms in females may also relate to the assumption that men are more at risk of cardiovascular disease than women [58].

Within the general cardiovascular literature, there is little awareness or prioritisation of cardiovascular disease by women. A 2018 survey by the Australian National Heart Foundation found that compared to 55% of men, only 39% of women aged > 45 years in Australia had undergone a heart risk assessment by a health professional in the prior 2 years [59]. Additionally, physician bias contributes to less cardiovascular disease screening and reduced primary and secondary prevention in women than men. An Australian study showed that women were 12% less likely to be screened for cardiovascular disease in general practice than men. Women were, therefore, less likely to have risk factors assessed to allow overall risk to be determined [60]. However, following initiation of patient care at HCM referral centres, there is generally no sex-based differences in HCM-specific management [9, 12–14]. More work is required to elucidate the mechanisms contributing to the sex differences observed in HCM.

## Future Directions

Underlying these sex differences in HCM is a lack of sex-disaggregated research. Women have been consistently under-represented in studies representing only a third of published cohorts, including those informing clinical recommendations (Fig. 3). While this is likely representative of disease prevalence, ensuring a relatively equal sex representation is necessary to identify why these differences exist. Understanding the cause of these differences will only be possible if the evidence is presented for both sexes via pre-specified sex-disaggregated analysis. This requires planning sub-group analyses before data collection and enabling the collection, analysis and reporting of data separately for men and women, even if this is not the study’s primary focus. These data have the potential to inform research and patient care to the benefit of both sexes.

## Conclusion

There is overwhelming evidence that sex is vital in explaining some of the clinical heterogeneity seen among HCM patients. Better female representation coupled with sex-disaggregated analysis is essential to understanding these differences. Further understanding of the sex-specific interaction of genetics and the environment, especially the impact of reproductive health and sex hormones on HCM outcomes, will be largely beneficial.
